# Breast Augmentation by Water-Jet Assisted Autologous Fat Grafting: A Report of 300 Operations

**DOI:** 10.1055/s-0036-1584165

**Published:** 2016-05-17

**Authors:** Daniel P. Muench

**Affiliations:** 1 Practice for Outpatient Surgery, Wiedlisbach/Berne, Switzerland

**Keywords:** breast augmentation, fat transplantation, autologous fat graft, liposuction, WAL, lipofilling

## Abstract

**Background** The BEAULI -method (Breast Augmentation by Lipotransfer) is available for extraction and processing of large transplantable fat quantities. The aim of this work is to describe the surgical technique precisely and reproducibly and to provide an overview of the autologous fat transfer based on surgical experience.

**Method** The author performed 300 autologous fat transplantations on 254 women between September 3, 2010, and May 13, 2015. Patients desiring moderate volume increase, fuller and firmer breasts, as well as an optimization of the silhouette, ideally with the concurrent desire of the correction of unwanted fat deposits, were selected. The fat was extracted via water-jet assisted liposuction (Body-jet, Human Med AG, Schwerin, Germany), and the fat cells were subsequently separated with the Lipocollector ^®^ (Human Med AG, Schwerin, Germany).

**Results** The results were assessed with a control exam and photo comparison and were based on the responses on a questionnaire. Overall, 35.9% of the patients defined the result as very good, 38.6% as good, 22.4% as satisfactory, and 3.1% as poor.

**Conclusion** This study shows that the autologous fat cell transplantation into the female breast via water-jet assisted liposuction achieves a moderate and harmoniously appearing breast volume enlargement as well as contour improvement. Further studies with more cases and longer observation periods over several years could contribute to improving the method of the autologous fat transfer regarding the grow-in rate, efficiency, and safety.

Autologous fat transfer for breast augmentation is gaining more acceptance as an alternative to implants because only autologous filling materials are used and because undesirable fat can be removed from other regions as an additional benefit. The advantages of breast augmentation with autologous fat include the lack of reactive inflammation, foreign body reaction, and scars as well as the natural-appearing result with more volume, an improved contour, a fuller cleavage, and natural consistency.

The competence and the experience of the surgeon, the technique of the extraction and processing of the fat, as well as the fat cell injection method are critical points regarding patient safety and result. Other factors are patient selection, the efficiency of the liposuction, the tumescent anesthesia, the instruments, a minimal blood loss, a high rate of survival of the transferred fat cells, and a minimal complication rate. 1 The literature varies regarding stem cell enrichment, grow-in rate, durability of the result, complications, limitations of future preventive breast exams, and possible tumor induction. Ross et al concluded in their research of 103 publications that there is no consensus regarding the optimum technique. 2

This single-surgeon study addresses the practical aspects of the gentle and rational fat recovery via water-jet assisted liposuction, fat processing, as well as the reinjection of the adipocytes. Exactly the same technique is used by the same surgeon with a high caseload so that the results can be considered reproducible.

## Patients and Methods

The author performed autologous fat cell transplantation for breast augmentation in 254 women between September 3, 2010, and May 13, 2015. Forty patients were operated twice, and two patients, three times. Three hundred procedures were performed in total ( Table 1 ). Women desiring a moderate and naturally appearing volume increase of the breasts, ideally also desiring a correction of unwanted fat deposits, were considered ( Table 2 ). Contraindications were uncontrolled diabetes, cardiovascular diseases, autoimmune disorders, infections, and anticoagulation therapies. The surgery was chosen by 40% of the patients based exclusively on the desire for a better breast shape. 51% of the women wanted a liposuction as well as a breast augmentation, and in 9% of the patients there was the primary desire for a liposuction. A volume asymmetry was corrected in 70 surgeries, and in 15 cases, only one breast was augmented. The success was judged based on the before-and-after comparison of photos taken in a standardized manner and on the subjective statements of the patients during the follow-up exam and their answers on a questionnaire. The average follow-up was 24.5 months (0.5 to 56 months). The patients were informed preoperatively about the risk of calcifications as well as the volume gain to be expected in the sense of firmer-appearing breasts (∼100 to 150 mL volume increase or approximately one-half cup size). A preoperative mammography and/or sonography was performed in patients above the age of 40 years. 3 Photos of the breasts from a frontal and lateral perspective documented the preoperative findings. The procedures occurred under perioperative antibiosis with 2 × 500 mg cefuroxime as well as prophylactic low-dose heparinization.

**Table 1:** Patient demographics, surgeries

Demographic	
Female patients ( *n* )	254
Patients operated one time ( *n* )	214
Patients operated two times ( *n* )	40
Patients operated three times ( *n* )	2
Total number of surgeries ( *n* )	300
Median age, years (range) ± SD	35.8 (17–67) ± 9.7
Median weight, kg (range) ± SD	62.3 (46–98) ± 8.8
Median BMI (range) ± SD	22.5 (17.1–32.3) ± 3.01
Donor region for the liposuction (%)	
Abdomen	60
Hip/groin region	59
Thigh	74
Fatty tissue transfer	
Average filling volume on the right, mL (range) ± SD	212 (70–350) ± 57.7
Average filling volume on the left, mL (range) ± SD	202 (70–340) ± 61.3
Average filling volume total, mL (range) ± SD	414 (140–690) ± 60.5
Surgeries with uneven filling volumes in the event of slight asymmetry ( *n* )	70
Surgeries with one-sided filling in the event of severe asymmetry ( *n* )	15
Average time of the surgery, min (range), SD	113 (80–165) ± 20.0

Abbreviations: BMI, body mass index (kg body weight/m ^2^ ); SD, standard deviation.

**Table 2:** Indications

Implants	Autologous fat transfer
Desire for large volume increase	Desire for moderate, harmonious volume increase and a contour improvement; “gentle” and naturally appearing breast forming
Foreign body does not matter	Aversion against foreign material
Smoker	Nonsmoker
BMI < 18.5	Normal weight or slightly overweight
Refusal of a liposuction	Concurrent desire for the correction of the body proportions

Abbreviations: BMI, body mass index (kg body weight/m ^2^ ).

### Infiltration and Lipoaspiration

All procedures were performed in an ambulatory setting with patients under local anesthesia and under sedation with 7.5 mg Midazolam (Dormikum ^®^ , Roche, Basle, Switzerland) taken orally and additional sedation with a gas mixture of 50% nitrous oxide and 50% oxygen (Entonox ^®^ , The Linde Group, Munich, Germany). The patient retained consciousness and could turn into an optimum position by herself, which made the liposuction easier to perform.

The anesthesia fluid preheated to 37°C was distributed in the subcutaneous fat layer in a fan-shaped manner through an opening with an angle of 30 degrees longitudinally at the end of the cannula. The solution was infiltrated with a pulsating flow of 110 mL/min and a pressure of 50 bars. The cannula was slowly moved back and forth in a fan-shaped manner following the pulsating jet spray and turned around the longitudinal axis at the same time. This allowed for a painless and gentle infiltration of the entire subcutaneous fat layer. Preinfiltration was beneficial for very sensitive patients, where only small volumes of the solution were first infiltrated into the marginal area of the suction zones (“ring block”) and in sensitive areas (bony prominences, belly button). The tissue was not firmly filled as in the typical tumescent technique (which could damage the fat cells due to pressure or osmosis), but instead this technique entailed a basic infiltration of the subcutaneous fatty tissue. The infiltration at the breasts started in a ring-shaped manner around the breasts and then the subcutaneous and subglandular layers were preinfiltrated.

**Fig. 1:**
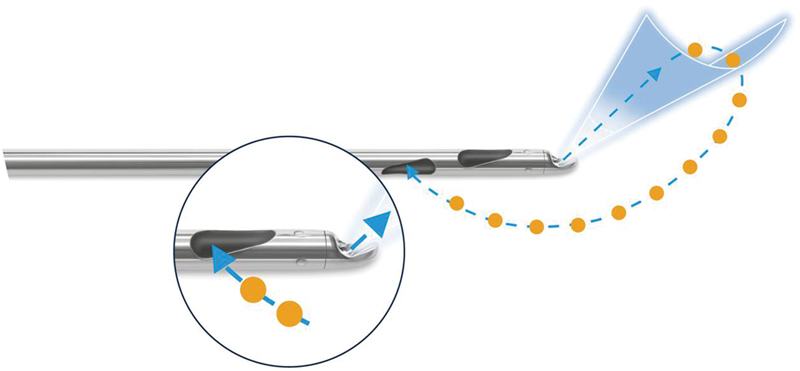
Cannula for simultaneous irrigation and aspiration (Human Med AG, Schwerin, Germany). The adipocytes are gently released by water, not fragmented or liquefied by physical power of the cannula.

Suctioning was performed with a double-lumen cannula with a length of 25 cm and a diameter of 3.8 mm ( Fig. 1 ). The anesthesia fluid was distributed in the tissue in a fan-shaped manner at the tip of the cannula analogous to the preceding infiltration phase. Four lengthwise oval suctioning openings arranged laterally/circularly in a distance of 25 mm to the tip of the cannula and with an effective diameter of 0.9 mm enabled the recovery of sufficiently small fat cell complexes so that these did not clog the 2-mm fat filling cannulas. The pressure was limited to a maximum of −0.55 bar to handle the fat cells as gently as possible. The adipocytes were flushed out of their extracellular matrix with a pulsating water jet (pressure 30 bars, flow 90 mL/min) under slow, uniform back-and-forth motions and were gently aspirated. Table 1 lists the distribution of the donor regions.

### Fat Processing and Reinjection

**Fig. 2:**
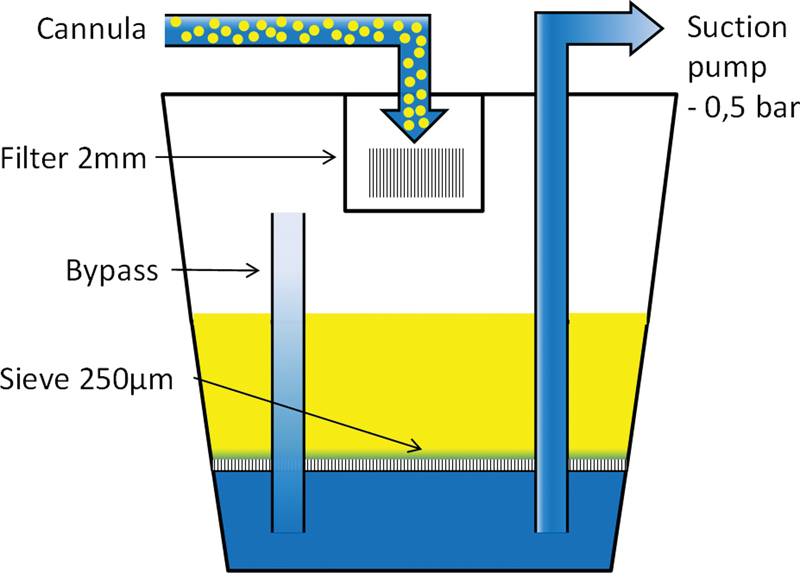
Schematic view of the Lipocollector (Human Med AG, Schwerin, Germany). A sieve with 2.0-mm narrow slits filters coarser connective tissue structures and larger fat particles from the aspirate, which have a poor grow-in rate. A fine filter with a pore diameter of 250 μm separates the harvested fat cells from the excess liquid.

**Fig. 3:**
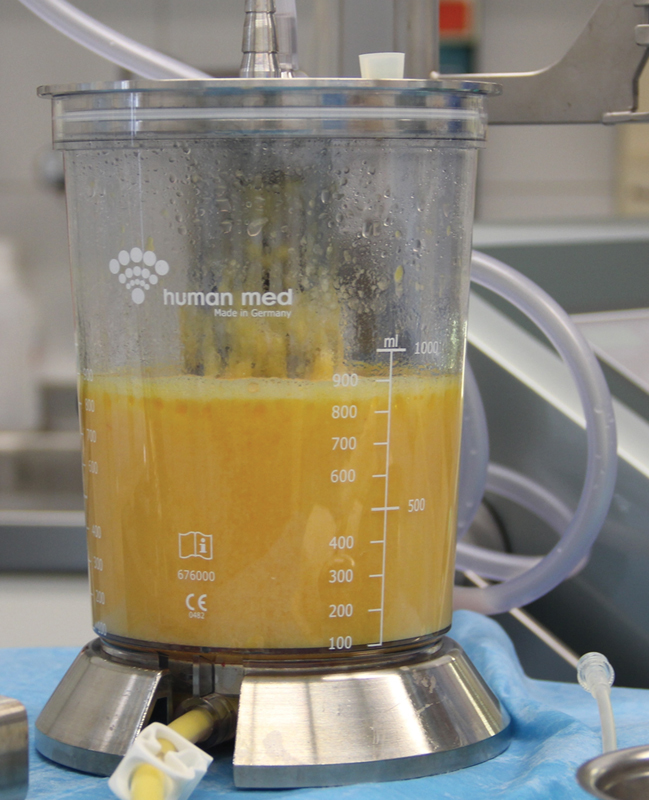
Lipocollector ^®^ (Human Med AG, Schwerin, Germany) after completion of the liposuction.

**Fig. 4:**
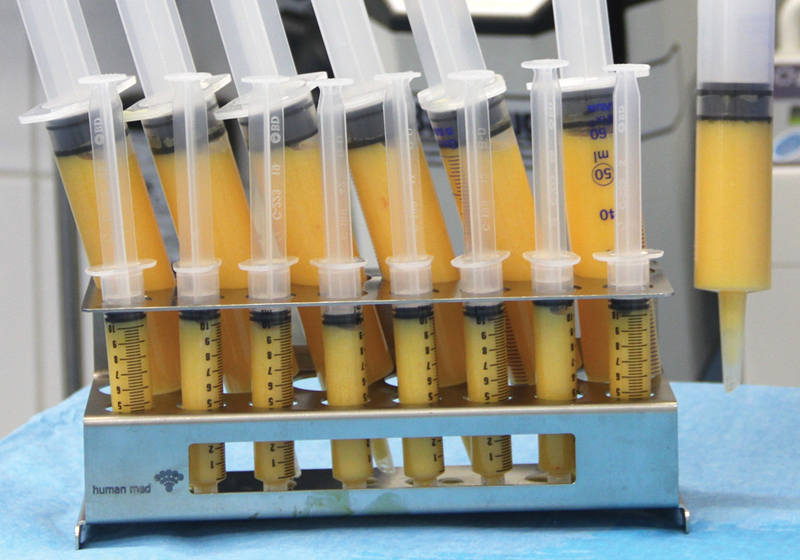
The pure transplantable fat, ready for reinjection, is transferred into syringes. It takes on average 60 minutes to harvest 600 mL of fat tissue.

The aspirate was collected by the interconnected, sealed, and sterile collector. A sieve with 2.0-mm slits filtered the coarser connective tissue structures and larger fat particles from the aspirate, which have a poor grow-in rate. A fine filter with a pore diameter of 250 μm separated the harvested fat cells from the excess liquid. Fig. 2 shows a schematic of the collector. Fig. 3 shows the conclusion of the liposuction. A residual amount of ∼15% liquid remained; this dilution is desirable as it allows for a gentle reinjection of the fat cells and supports their even distribution in the host tissue. After the conclusion of the liposuction, the adipocytes were transferred in a sterile manner without contact to air into first 60-mL and then 10-mL syringes ( Fig. 4 ).

**Fig. 5:**
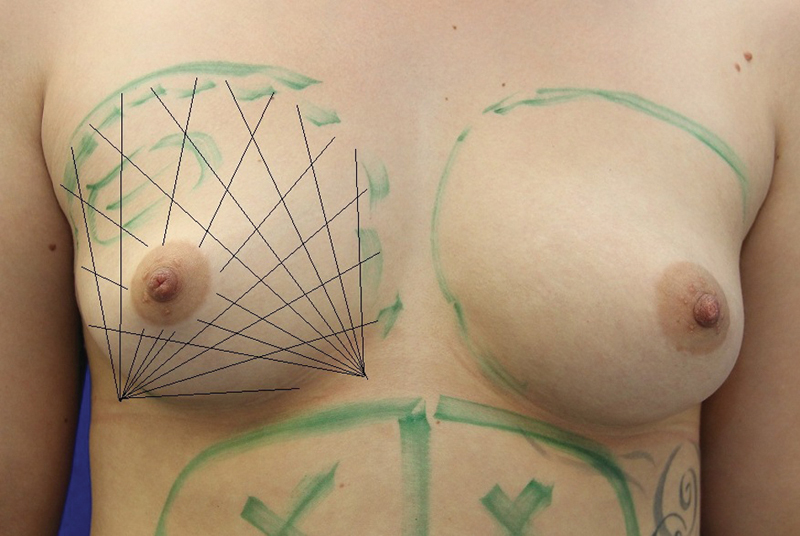
The reinjection of the fat cells is implemented from two incisions in a fan-shaped manner and in all layers. The infiltration occurs via a gentle, even pressure onto the syringe plunger while rotating and pulling the cannula back at the same time. The tissue must not be “overfilled” to protect the fat cells from an additional pressure.

The fat cells were reinjected through two small lateral caudal and parasternal stab incisions with a respective distance of 2 cm to the breast ( Fig. 5 ). A 2-mm cannula (12 gauge) with a length of 150 mm and two lateral openings at the end of the cannula was used. The infiltration exclusively occurred in the subcutaneous and subglandular fat layer as well as in the area of the pectoralis muscles but never intraglandularly. The upper body of the patient was angled by 45 degrees for the infiltration and the arms rested on the side of the body. The tissue was less tense this way and the cannula could be guided more easily and more precisely. The infiltration occurred via a gentle, even pressure onto the syringe plunger while pulling the cannula back at the same time. The cannula was thereby continuously rotated from left to right. The adipocytes were thus distributed linearly like a string of pearls and in fine portions. The entire breast including the neckline was evenly infiltrated in a fan-shaped manner and in all layers (“three-dimensional filling”) from the two stab incisions. The goal was a honeycomb structure of microtunnels with a thickness of 2 to 3 mm. This approach is required so that the diffusion distance is as small as possible and so that as many fat cells as possible can connect to a blood vessel. The tissue must not be “overfilled” to protect the fat cells from an additional pressure. The volume was around 100 to 120 mL in small, firm breasts and 250 to 300 mL in larger, rather flaccid breasts. The mean value of the infiltrated volume was 207 mL per side (70 to 345 mL) in our series. The average time of the surgery (including liposuction) was 113 minutes (80 to 165, ± 20.0 standard deviation).

## Results

**Fig. 6:**
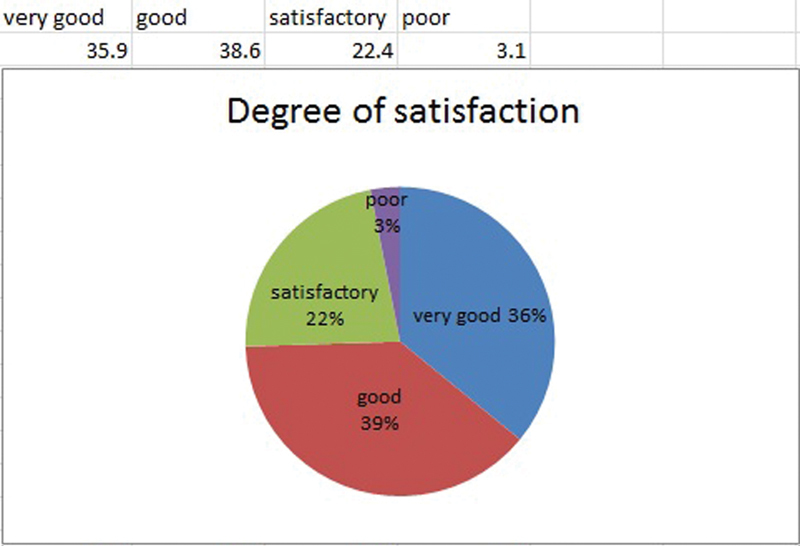
Degree of satisfaction.

**Fig. 7:**
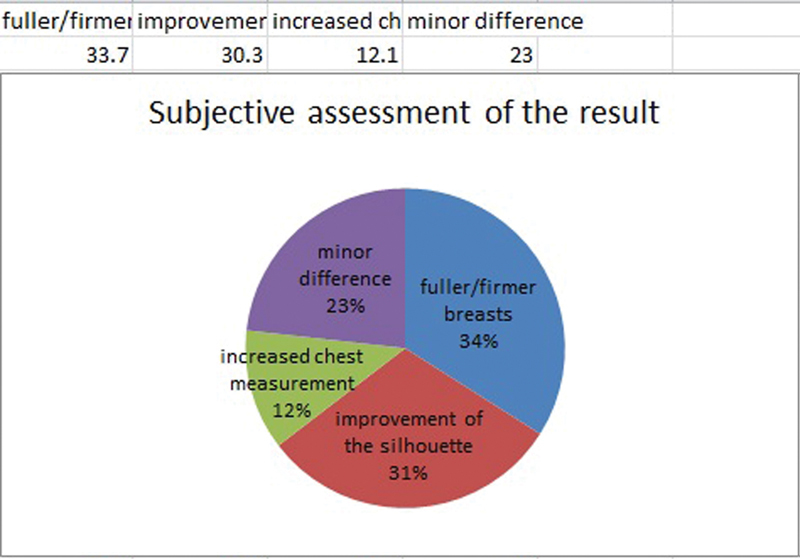
Subjective assessment of the result.

**Fig. 8:**
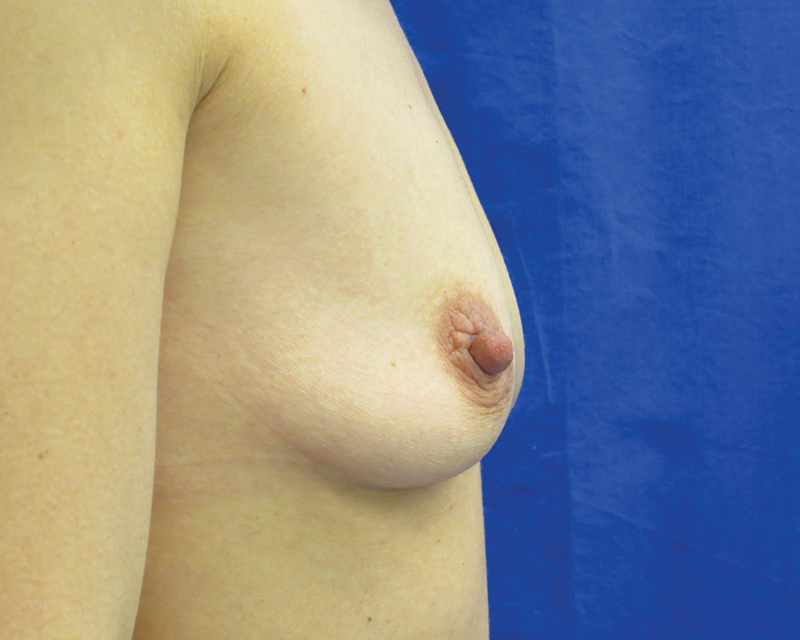
A 35-year-old patient, preoperative view.

**Fig. 9:**
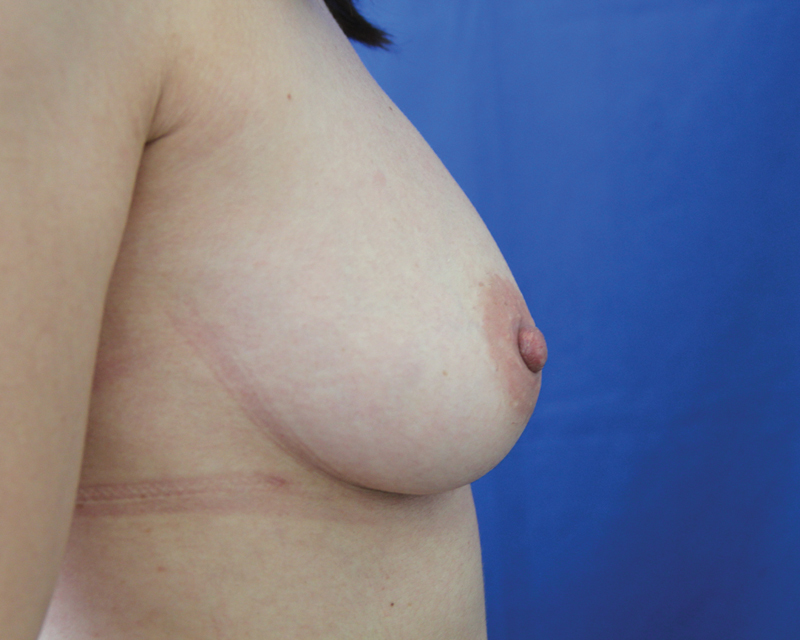
Patient in Fig. 8 after two sessions of breast lipofilling, 6 months after the last procedure (injections of 240 and 150 mL, respectively).

**Fig. 10:**
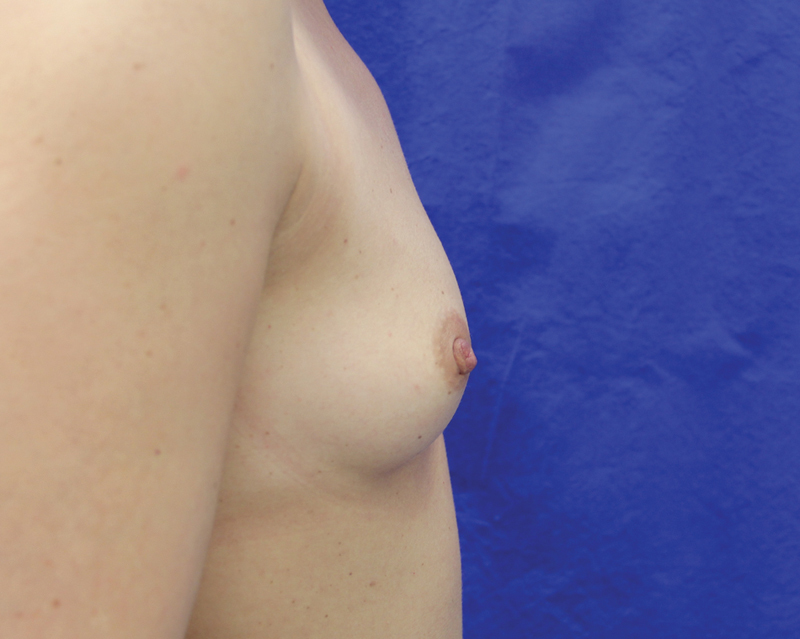
Preoperative view of a 39-year-old woman requesting breast augmentation.

**Fig. 11:**
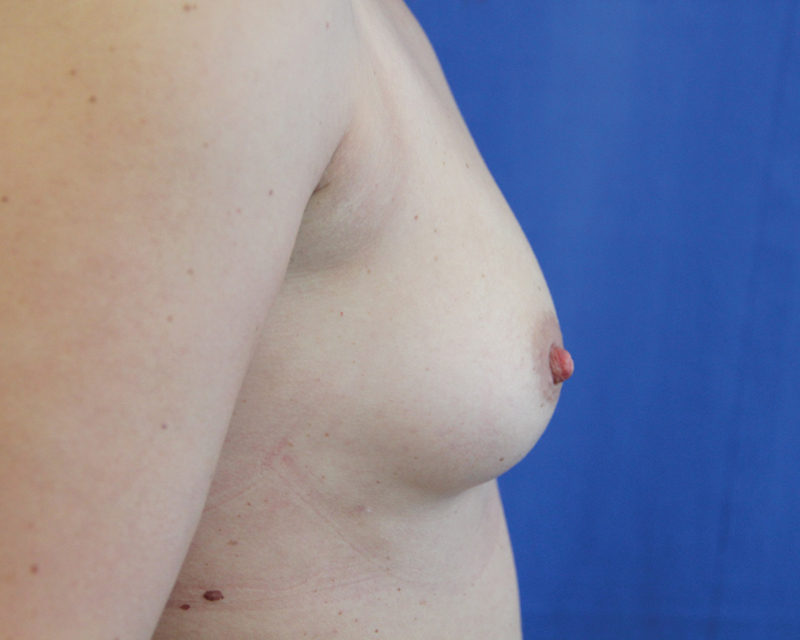
Postoperative view of patient in Fig. 10 at 1 year after breast augmentation with 280 mL fat in each breast.

**Fig. 12:**
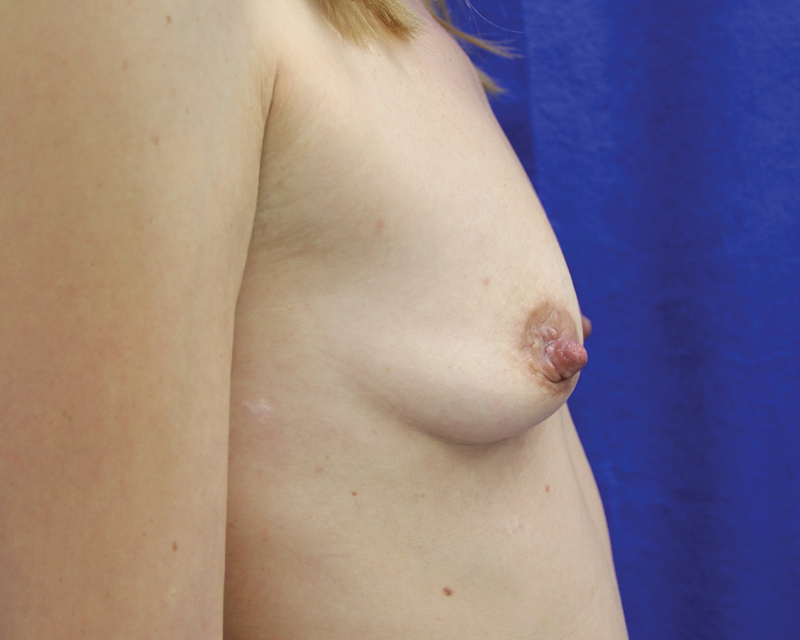
Preoperative photos of a 38-year-old woman.

**Fig. 13:**
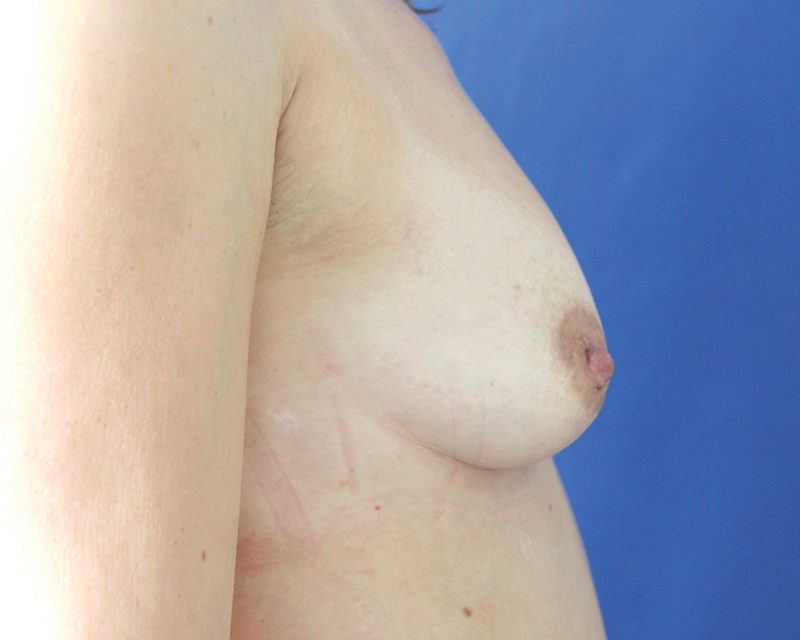
One-year postoperative view of patient in Fig. 12 following breast filling with 240 mL in each breast.

**Fig. 14:**
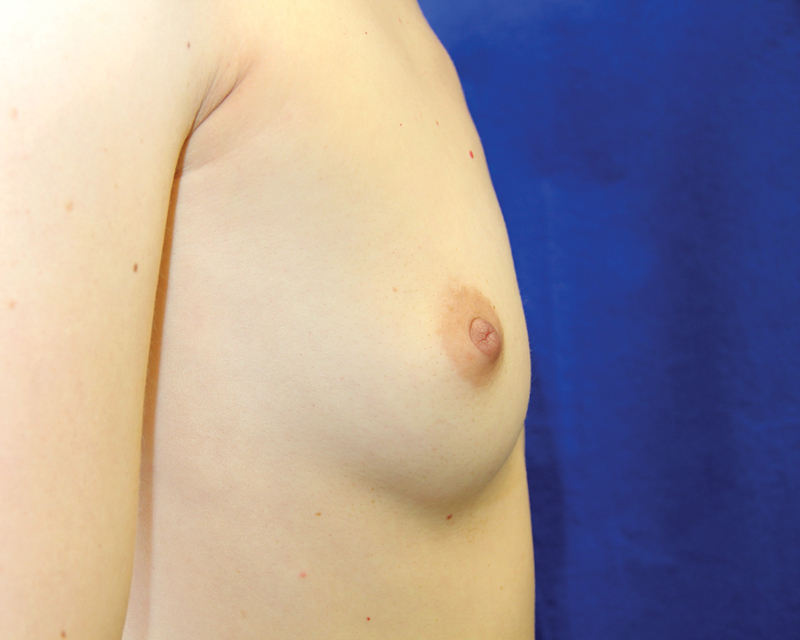
A 35-year-old patient, preoperative view.

**Fig. 15:**
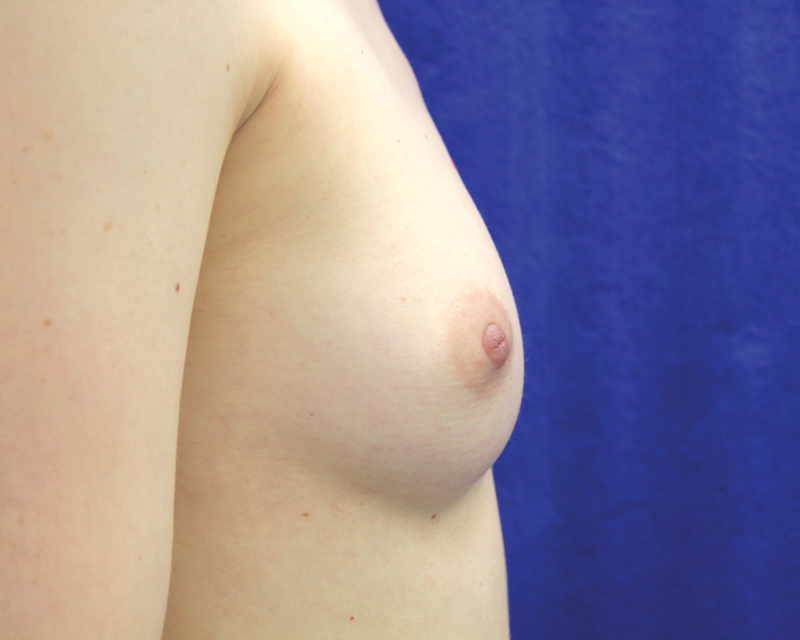
Postoperative photos of patient in Fig. 14 at 1 year and 6 months after breast augmentation with 250 mL in each breast.

**Fig. 16:**
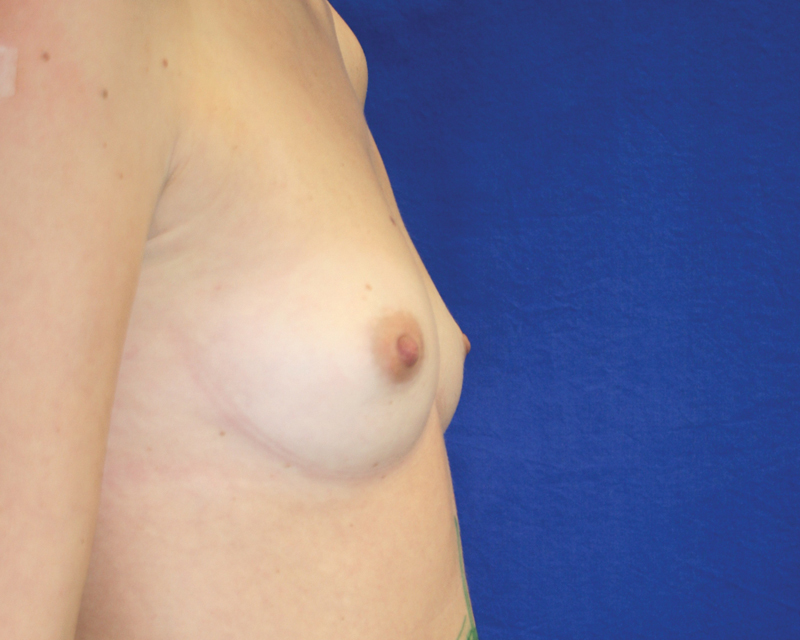
Preoperative view of a 27-year-old patient.

**Fig. 17:**
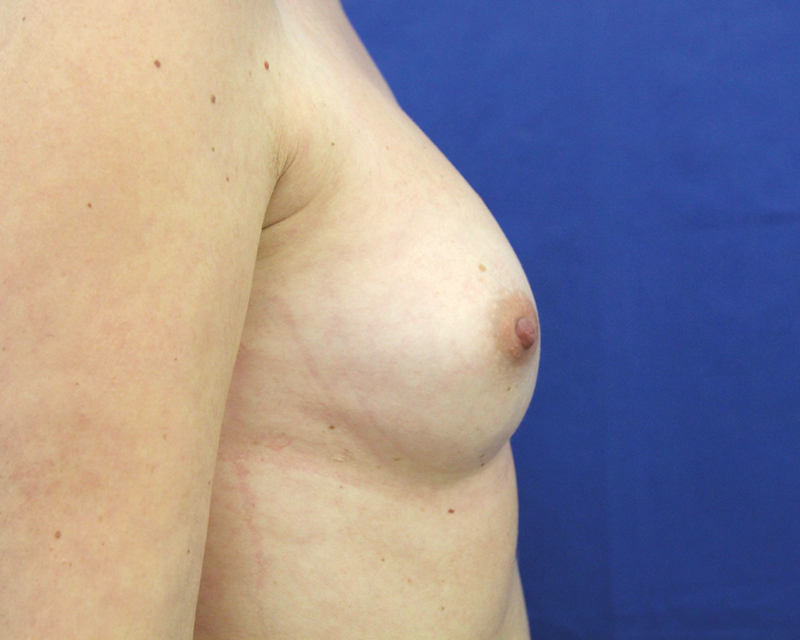
Patient in Fig. 16 after two operations of breast augmentation (filling of 140 and 250 mL, respectively), with no more volume loss even 2 years after the last session.

**Fig. 18:**
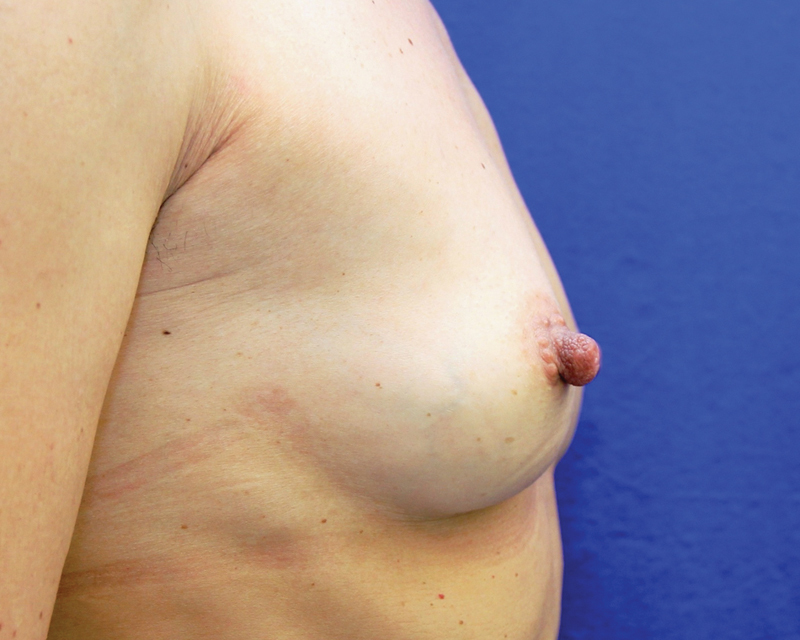
Preoperative view of a 52-year-old patient.

**Fig. 19:**
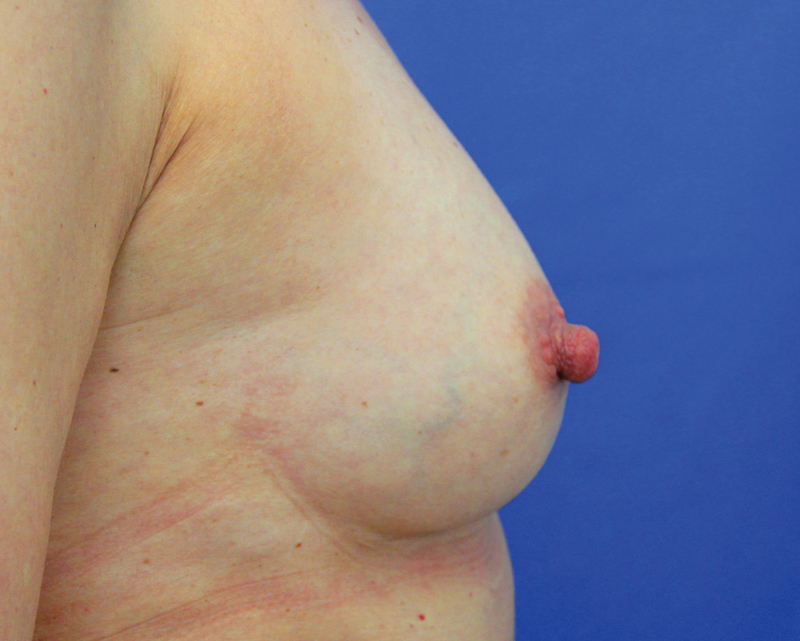
Postoperative photo of patient in Fig. 18 after breast augmentation with 220 mL fat in each breast with satisfactory result even 3 years und 3 month after the operation.

Very good to good results in improvement of contour and increase in volume were achieved in 74.5% of the cases. The results were assed as very good by 35.9% of the patients, as good by 38.6%, as satisfactory by 22.4%, and as poor by 3.1% ( Fig. 6 ); 33.7% of the patients subjectively identified the success primarily as a feeling of fuller and firmer breasts, 30.3% named the improvement of the silhouette first; 12.1% primarily described an increased chest measurement; and 23% noticed only a minor difference ( Fig. 7 ). Mainly elderly patients fall into the minor difference category, where only small amounts of autologous fat could be extracted, or when patients lost weight in the meanwhile. Objectively, there was a mostly clear difference to be seen in the before-and-after photos in all cases of patients undergoing a follow-up exam ( Figs. 8 to 19 ). Overall, 39.4% would undergo another transfer surgery to achieve even more breast volume, 29.5% were uncertain, and 31.1% did not want another procedure.

Postoperative complications such as infections, necrosis, hemorrhages, seromas, or thromboembolic events did not occur after any of the 300 surgeries. Other than the usual swelling and tenderness on palpation, temporary redness or skin irritations were reported only rarely, and postoperative subcutaneous hematoma was observed in 13 cases (4.3%); all of these findings disappeared within 6 months. Contour irregularities occurred in 6 cases (2%) and asymmetries in 5 cases (1.6%). In the postoperative phase, 36.1% of the patients defined the pain as low, 47.2% as bearable, and 16.7% as rather severe. The incapacity to work (office work) after the procedure was up to 2 days in 45.7% of the cases, between 3 and 4 days in 40.3%, and between 5 and 7 days in 14%.

## Discussion

The method of the autologous fat cell transplantation for the correction of body defects or for cosmetic indications developed from liposuction for the correction of the body proportions. Various methods of extraction, processing, and transfer have been described. The effectiveness and healing rates are also a topic of discussion. Fournier was able to demonstrate that intact adipocytes can be harvested via syringe aspiration. 4 Coleman proved that with the lipostructure method it is generally possible to transplant autologous fat cells successfully. 5 However, his method is very time-consuming: a procedure takes 6 to 8 hours. Li reported in 2014 more than 105 surgeries of autologous fat transfers into the female breast, whereby a significant increase in volume and an improvement of the form was achieved in all cases. 6

The goal of an autologous transfer is to achieve a maximum, sustainable volume increase in the host area. Unprocessed fat contains three main components: intact fat cells, liquefied fat, and serosanguineous fluid. These components must be separated to achieve an optimum concentration of intact adipocytes without any blood or oil mixed in. Fat cells do not tolerate excessive manipulations such as supercooling, freezing, mechanical stress, or substantial pressure fluctuations. Traumatic fat extraction, incorrect cannulas, too much negative pressure, blood mixed in, exposure to air, and contamination are possible causes for a failure. The age of the patient, genetic dispositions, and smoking and nutritional habits also have an impact on the result. 7 8

A prerequisite for the survival of fat cells is that the surface-to-volume ratio is as large as possible. Small, drop-shaped transplants have a relative large surface in comparison with larger fatty tissue pieces. This accelerates the vascularization and leads to a better rate of resorption. To achieve a high rate of survival of larger transplant volumes, large quantities of sufficiently small fatty tissue packages must be distributed absolutely evenly and finely in the well-perfused host tissue. The survival of transferred fat cells prior to the connection to a blood vessel depends on diffusion. It can be assumed that fatty tissue pieces with a maximum diameter of 1 mm will grow in. 9 It takes between 24 hours and 4 days for the angiogenesis around the fat particles to begin. 5 10 It is therefore important that no pressure is exerted onto the host area and that the body temperature does not drop. The extracorporeal time of survival of adipocytes is 4 to 6 hours at moderate temperatures, and longer surgery times should therefore be avoided. 11

### Anesthesia and Thrombosis Prophylaxis

Autologous fat transfer is nowadays performed under local anesthesia. The question if general anesthesia should be preferred due to the potentially damaging effects of lidocaine on the cell vitality is not clearly answered. 12 13 Shiffman recommends a general anesthesia or a regional anesthesia because the Klein solution is unphysiologic and the fat cells absorb fluid due to the oncotic gradient. 10

The early mobilization, the use of compression clothing, the hemodilution effect of the Klein solution, and the thrombocyte aggregation-inhibiting effect of lidocaine are factors that support the thrombosis prophylaxis. Individual cases of pulmonary embolisms have been described in the literature. 14 A thrombosis prophylaxis with low-dose heparin (20 mg per day, subcutaneously) appears to be indicated for fat cell transfer into the breast. The question if heparinization can improve the rate of resorption remains open.

### Donor Zone

The wishes of the patient are primarily followed regarding the donor zone. Chiu concluded that even a body mass index below 18.5 does not present a contraindication for a fat transfer. 15 Rohrich et al compared fat extracted with liposuction from different donor zones and did not find differences in regard to the viability of the fat cells. 16 Small et al also concluded that the grow-in rate is not affected by the donor zone. 17 If the transplant originates from “primary fat depots,” so from diet-resistant or genetic zones, then it can be assumed that this characteristic is also maintained in the host zone. Fat cells with α2 receptors are antilipolytic and react poorly to diets. These adipocytes react less to future weight fluctuations and appear to have a better rate of survival than adipocytes with β1 receptors. 18 Studies show that the breast volume increased disproportionately in patients gaining weight after an autologous fat transfer from diet-resistant zones and the liposuctioned zones only exhibited minor changes. 13

### Tumescent Solution

The possible damaging metabolic and inflammatory effects of lidocaine on the donor fat and the host zone are not clear. 13 Lidocaine can inhibit the glucose transport of cell cultures in vitro in concentrations from 0.05 to 0.1%. Moore et al, however, observed this effect only in the event of direct contact of the fat cells to the anesthetic. 19 The same authors postulated that these potentially unfavorable effects on the cell viability can only be observed during the effective period of the local anesthetic (lidocaine: 1 to 3 hours) and additionally is reversible. Keck et al analyzed human preadipocytes previously treated with different local anesthetics and NaCl. 12 The direct effects of mepivacaine and ropivacaine were moderate. However, increased damage was observed in cells treated with articaine/epinephrine. Lidocaine is therefore still the preferred local anesthetic with a vitality rate of 80% (in comparison with 90% in the NaCl control). Keck et al analyzed the effect of the pH value of the substances of the tumescent solution on the cell viability of the adipocytes and concluded that the viability in the buffered solution is up to 11% higher than in the nonbuffered solution. 12

“Blood is the enemy of fat” 20 : blood mixed in with the transplant can lead to an inflammatory reaction of the fatty tissue and stimulate the macrophage activity. Cells are resorbed as a result. Blood can also inhibit the neovascularization and is a good culture medium for the growth of bacteria. The addition of adrenaline to the Klein solution is therefore imperative. Adrenaline as a vasoconstrictor does not appear to affect the viability of the fat cells. 19 The capillaries of the fatty tissue are extremely sensitive to epinephrine; the addition of adrenaline to the Klein solution therefore leads to a distinct vasoconstriction and thus efficiently stops the bleeding.

### Liposuction

By means of water-jet assisted liposuction (WAL), the fat is gently flushed out of the tissue by the water jet, which leads to a higher viability of the lipoaspirate and improved rates of survival of the fat cells in comparison with common liposuction methods. 21 Our cannulas have a 0.9-mm opening, which allows us to harvest adipocytes atraumatically and in sufficiently small particles without clogging the cannula. Histologic analyses showed that the membranes of the cells rupture and fat cells evaporate with suction stronger than −0.5 bar. 22 We limit the pressure during the aspiration to −0.5 to −0.55 bar. An even lower negative pressure clogs the suction cannula and reduces the efficiency of the fat recovery. 3 The fat should also not be exposed to abrupt pressure fluctuations, for example, when the liposuction is interrupted. The aspirate should not come into contact with air, which can impair the quality of the adipocytes. 14

### Fat Processing

The goal of the processing is to concentrate the aspirate regarding adipocytes, growth factors, and stem cells and to separate these from blood, serum, and destroyed fat cells. Some authors propagate the centrifugation or the sedimentation of the aspirate for the separation of water, blood, and free lipids. 14 20 However, excess centrifugation can destroy the adipocytes and the fat stem cells. In the BEAULI-method (Breast Augmentation by Lipotransfer), the fat cells are concentrated sufficiently via the water jet technique and functioning of the collector with filter and sedimentation effect so that centrifugation (and its damage to the cells) is not necessary. The traumatizing effect on the sensitive fat cells and the fact that washing destroys the microenvironment of the cells (collagens, vessels, proteins, proteases, enzymes, electrolytes) speaks against washing the aspirate; also, the fibrin, which stabilizes the adipocytes in the wound site, is flushed out. 23

### Bioactivators

The Celution ^®^ System (Cytori Therapeutics, San Diego, California, United States) is supposed to be able to isolate stem and regenerative cells from the fatty tissue. However, Peltoniemi et al proved that this method of the stem cell enrichment does not show any advantages over the WAL fat recovery method but does incur additional expenses, loss of time, and an increased risk of infection. 24 McArdle et al concluded that stem cell procedures are to be rejected in aesthetic surgeries due to the lack of clinical evidence and the risk for the patient. 25

Yoshimura et al described the method of the cell-assisted lipotransfer. 26 Their reflections are based on the observation that aspirated fat contains fewer vessels than intact fat and has a deep concentration of adipose-derived stromal cells/stem cells (ASCs). The stromal vascular fraction (SVF), which includes the ASCs, is isolated from one-half of the recovered fat via enzymatic collagen digestion reactions of the extracellular matrix, centrifugation, and electrolysis. This fraction is then mixed with the other half of the aspirated fat. This process makes it possible to harvest ASC-rich fat from a relatively ASC-poor fat aspirate. Tabit et al found that the additions of ASCs recovered from the SVF could promote the grow-in rates and the survival of fat transplants. 27 Brayfield et al described the possibility of isolating ASC from the human fatty tissue, which can develop into pure adipocytes. 28 The authors especially pointed out the angiogenic potential of the ASCs and emphasized the significance of these cells in the area of transplantation. Philips et al concluded that the ASCs will play an important role in grow-in rate improvements in autologous fat transplantations. 29 Multiple cells can be typified from the SVF based on the surface cell markers—in addition to the ASCs already mentioned, also endothelial progenitor cells and pericytes. Largo et al studied the SVF in regard to cell quality and composition and demonstrated a negative correlation between the age of the patient and the relative progenitor cell count, 14 which could indicate a reduced vascularization potential or a poorer grow-in rate. The authors concluded that the endothelial progenitor population of the freshly isolated SVF or the lipoaspirate could support the neovascularization of the transplanted fat cells. Ueberreiter et al postulated that stem cell enrichment does not promote the grow-in rate of the adipocytes but rather the proximity to a blood vessel is decisive. 3

A potential problem in breast enlargement is that the fat cells serve as expanders and as natural tissue filler at the same time. The tension in the tissue can impair the blood flow in the host, can cause local ischemia, and thus can reduce the grow-in rate. Khouri and colleagues therefore recommended to expand the tissue with the nonsurgical BRAVA ^®^ -system (Taureon Ltd, London, United Kingdom) beforehand. 30 31 However, this technique can also lead to pain, edema, and tissue irritations, and in addition, the compliance is to be classified as rather low.

### Cryoconservation of Fat

Cryoconservation of fat would be desirable to reduce the number of liposuctions in the event of a repeated fat transfer. Raskin found great differences in regard to the temperature (+4°C to −70°C, slow or fast freezing and thawing) and the duration of the conservation (up to 3 years) in his literature research. 32 The studies analyzed the conserved fat in regard to changes in volume, weight, histologic parameters, viability, stem cell concentration, bacterial contamination, and clinical courses. The data in the literature allows for the conclusion that a cryoconservation of the fat cannot be considered due to the possible complications and the reduced resorption rates. Matsumoto et al recommended to infiltrate the aspirated fat as soon as possible; they found an increase in the oil volume within 4 hours at room temperature. 11

### Reinjection

The infiltration is a decisive factor for a successful fat cell transplantation. No matter how good the quality of the fat cells is, if the transplant is distributed unevenly, in clumps, in incorrect layers, or with an excess pressure during the final infiltration, then failure is certain. The diameter of the infiltration cannula should be at least 18 gauge as the metabolic activity of the particles can be impaired by the pressure during the injection otherwise. We work with cannulas with a length of 150 mm, a diameter of 2 mm, and two lateral openings offset by 180 degrees with a diameter of 1.2 mm. This guarantees that the fat particles are distributed evenly and with minimal trauma to the host tissue and that the cannula does not become clogged. The fat particle injections occur in the subcutaneous, subglandular, and interpectoral fat layer, never in the mammary gland tissue. Herold et al found a volume preservation of 81% in the periglandular fatty tissue and a volume preservation of 64% in the muscle at 6 months after the fat cell transplant in a MRI-volumetrically measured series. 33 The pectoralis muscles are thus a possible additional target volume to increase the safely injectable total volume.

It is not possible to improve the final volume increase by an overfilling; overfilling results in necrosis and overgrafting results in loosing fat. The additional pressure generated thereby can impair the blood flow, destroy fat cells, and thus increase the risk for the formation of cysts and calcifications. If a greater volume increase is desired, then it is recommended to proceed in several visits in at least 4-month intervals with a lower transplant volume. Hoppe et al recommended four to six lipotransfers over a period of 2 years for a total breast reconstruction. A patient satisfaction of 95% as achieved in 28 cases this way. 34 Harder et al repeated the fatty tissue transfer up to four times. 35

### Results

Success was based on the subjective statements of the patients as well as the before-and-after comparison of photos taken in a standardized manner. Volumetric evaluations via magnetic resonance imaging (MRI) appear to be the superior method regarding the concurrent verification of structural changes and the accuracy of the results. These measurements showed that the largest decline in volume occurred during the first 4 weeks, and only minimal changes were registered thereafter. Harder et al studied autologous fat transfer after breast surgeries and assumed that the resorption process is concluded after 4 to 6 months. 35

Ueberreiter et al performed autologous breast reconstruction in 85 patients with the WAL technique. 3 An MRI was performed preoperatively and in 72 cases at least 6 months postoperatively. The grow-in rate was 76%, resulting in a net volume increase of the breast of approximately 51.7% of the injected volume. Other authors used MRI to quantify the grow-in rate relative to graft volume with the WAL technique: Herold et al analyzed 10 patients and found an uptake of 77%, 33 and Peltoniemi et al studied 8 patients and found an uptake of 80%. 24 For comparison, various studies evaluated the grow-in rate after fat transfer with techniques other than WAL. In a study on fat transfer with preexpansion, Del Vecchio and Bucky reported an increase in volume of 106%, 36 relative to initial breast volume. A further study by Del Vecchio showed an uptake of 62%. 37 Khouri et al studied 71 procedures and found a graft survival of 82%. 38 Fiaschetti et al combined the fat transfer with platelet-rich plasma in 15 patients; the average uptake was 84%. 39 Largo et al determined the breast volumes with a three-dimensional laser scanner, 14 with fat survival rates varying between 25 and 90% even with the same surgeon and identical surgical techniques. Choi et al analyzed the grow-in rate after 140 days depending on the infiltrated volume. An infiltration of 151 mL resulted in a grow-in rate of 52%, and an infiltration of only 51 mL resulted in a grown-in rate of 27%. 40

### Complications

Poor grow-in rates can be the result of mixed-in blood, infections, incorrect extraction techniques, an excessive vacuum, fibrous donor zones, the injection of too-large transplant portions, or the use of destroyed adipocytes. Calcifications can occur as in any breast surgery. According to Largo et al, 14 the literature specifies the risk for calcifications and oil cysts to be up to 50%, and Ueberreiter et al described a frequency of 4.5%. 3 In a literature survey of 60 publications with 4,601 patients by Claro et al, 41 complications occurred in 3.9%, most of which were indurations and/or palpable hardenings. The multicenter study by Agha et al comprised 3,624 patients; fat necrosis occurred in 4.4% and biopsies were necessary in 2.7%. 42

The preoperative testing for white blood cell count, blood sugar, hepatitis, and HIV should exclude patients with an increased risk for infection. Active sources of infection (tonsils, abscesses) are contraindications for a fat transfer. Infections can be reduced via a strictly aseptic surgical technique, and the use of a closed suction and processing system without air contact. The infiltrate should be free of blood, and the surgery times should be within a reasonable range. Repeated disinfection of the injection site at each new insertion of the cannula, strict prevention of touching the cannula, and a prophylaxis with antibiotics offer additional safety.

### Preventive Breast Exams Using Imaging Methods and Tumor Induction

Microcalcifications, cysts, or bridles can be observed after any breast surgery and can be concomitant to the preventive exams. Veber et al could not demonstrate any significant differences in regard to calcifications and cystic lesions in a comparative study of mammographies after a breast filling. 43 Petit et al documented a deep complication rate and no limitations of radiologic preventive exams in a multicenter study with 646 lipofilling patients. 44 According to Coleman, calcifications do not occur more frequently during lipofilling than in other breast procedures. 45 Rubin et al found fewer radiologic abnormalities in 27 patients following autologous fat breast augmentation than in a comparison group with 23 patients. 46 In the literature compilation of Claro et al, 41 radiographic anomalies were observed in 13% of 2,560 women undergoing surgery, more than half of them in the form of cysts. Imaging techniques such as mammography, sonography, and MRI allow differentiation of calcifications in connection with malignant processes and benign calcifications caused, for example, by breast surgeries or filling procedures. 14 47 Kim et al sonographically detected fat necrosis and cysts in 17.6% of 102 patients, and the fat resorption rate was 32.9%. 48 So far, there has been no reported case of a delayed mammary carcinoma diagnosis due to calcifications after an autologous fat transfer. 49

No negative consequences, especially regarding the development of a carcinoma, have been described since the first publication of autologous fatty tissue transplantations more than 100 years ago, even after long follow-up. 3 Kronowitz et al identified patients who underwent mastectomy for breast cancer followed by reconstruction with and without lipofilling. 50 Recurrence was observed in 1.3% of 719 breasts with lipofilling and in 2.4% of controls without lipofilling. Rigotti et al compared the incidence of local and regional relapses of mammary carcinoma in 2010 in 137 patients who underwent an autologous fat breast reconstruction following a radical mastectomy. 51 After a mean observation period of 7.6 years, there was a comparable incidence of local relapses. In the compilation of Claro et al of 616 patients, 41 the rate of a local relapse in the breast reconstruction with autologous fat after ablation was 2.3%. Illouz and Sterodimas followed 230 patients after an autologous fat transfer into the breast for 11.3 years on average. 7 The annual mammograms did not show an increased incidence of breast lesions. Fraser et al did not find an increased carcinoma incidence in the review of several clinical studies with autologous fat transfer in more than 2,000 patients. 52 A meta-analysis of 35 studies with 3,624 patients after autologous fat transfer from the year 2015 did not show a significant difference in comparison with the control group in regard to the carcinoma frequency. 42 In accordance with these studies, the American Society of Plastic Surgeons in 2009 also came to the conclusion that the success of autologous fat transfer possibly depends on the technique and the experience of the surgeon. 53

## Conclusion

The transplantation of autologous, viable adipocytes and preadipocytes for the purpose of a contour improvement or a volume increase has become a routine procedure. Different methods of the fat recovery, processing, and injection are described in the literature. Further randomized studies with high case numbers, long-term follow-up, and the generation of standards to be able to reproduce the results as well as qualitative-quantitative analyses of the augmentation volume are necessary. The use of additives for the bioactivation of transplant and host tissue to increase the resorption rate is of substantial interest and must be studied further. All potential methods for the further optimization of the resorption rate should not affect the safety of the fat transfer of large volumes and should be practically feasible with reasonable effort.

Exogenous fillers such as silicone, collagen, and hydroxyapatite but also autologous materials such as skin flaps and local or free flaps have significant disadvantages and can lead to disappointing results. Some patients are afraid of the possible problems of the implants such as foreign body reactions, scars, asymmetries, pain, volume losses, capsule contractures, unevenness, or an unnatural appearance. The autologous fat cell transplantation into the female breast via is a good and safe method to achieve a gentle, harmoniously appearing breast volume enlargement as well as a sustainable and naturally appearing contour improvement. Selected and educated patients who have an aversion against foreign bodies and desire fuller breasts or a compensation of asymmetries are very satisfied with the results of this method, which is continuously gaining significance.
